# The Heritability of Glaucoma-Related Traits Corneal Hysteresis, Central Corneal Thickness, Intraocular Pressure, and Choroidal Blood Flow Pulsatility

**DOI:** 10.1371/journal.pone.0055573

**Published:** 2013-01-30

**Authors:** Ellen E. Freeman, Marie-Hélène Roy-Gagnon, Denise Descovich, Hugues Massé, Mark R. Lesk

**Affiliations:** 1 Maisonneuve-Rosemont Hospital Research Center, Montreal, Quebec, Canada; 2 Department of Ophthalmology, University of Montreal, Montreal, Quebec, Canada; 3 CHU Sainte-Justine Research Center, University of Montreal, Montreal, Quebec, Canada; 4 Department of Social and Preventive Medicine, University of Montreal, Montreal, Quebec, Canada; Vanderbilt University, United States of America

## Abstract

**Purpose:**

The purpose of this work was to investigate the heritability of potential glaucoma endophenotypes. We estimated for the first time the heritability of the pulsatility of choroidal blood flow. We also sought to confirm the heritability of corneal hysteresis, central corneal thickness, and 3 ways of measuring intraocular pressure.

**Methods:**

Measurements were performed on 96 first-degree relatives recruited from Maisonneuve-Rosemont Hospital in Montreal. Corneal hysteresis was determined using the Reichert Ocular Response Analyser. Central corneal thickness was measured with an ultrasound pachymeter. Three measures of intraocular pressure were obtained: Goldmann-correlated and corneal compensated intraocular pressure using the Ocular Response Analyser, and Pascal intraocular pressure using the Pascal Dynamic Contour Tonometer. The pulsatility of choroidal blood velocity and flow were measured in the sub-foveolar choroid using single-point laser Doppler flowmetry (Oculix). We estimated heritability using maximum-likelihood variance components methods implemented in the SOLAR software.

**Results:**

No significant heritability was detected for the pulsatility of choroidal blood flow or velocity. The Goldman-correlated, corneal compensated, and Pascal measures of intraocular pressure measures were all significantly heritable at 0.94, 0.79, and 0.53 after age and sex adjustment (p = 0.0003, p = 0.0023, p = 0.0239). Central corneal thickness was significantly heritable at 0.68 (p = 0.0078). Corneal hysteresis was highly heritable but the estimate was at the upper boundary of 1.00 preventing us from giving a precise estimate.

**Conclusion:**

Corneal hysteresis, central corneal thickness, and intraocular pressure are all heritable and may be suitable as glaucoma endophenotypes. The pulsatility of choroidal blood flow and blood velocity were not significantly heritable in this sample.

## Introduction

Most cases of open-angle glaucoma (including normal tension glaucoma) are probably multifactorial, involving multiple contributing genetic and environmental factors. Intermediate phenotypes, also called endophenotypes, are powerful tools in the search for genes contributing to multifactorial human diseases as they are likely to be more directly influenced by the genes than the resulting disease phenotype and they provide greater statistical power [1]-[4]. Phenotypes such as intraocular pressure and central corneal thickness may be suitable as endophenotypes in glaucoma since they are well-established risk factors for glaucoma [5], [6]. In addition, ocular elasticity may play a major role in the susceptibility of the optic nerve to glaucoma [7]. As the cornea and the sclera together form the external wall of the eye and are formed of a continuous tissue of extracellular tissue[8], the mechanical properties of the cornea might be a good marker for those of the entire wall of the eye. Therefore, corneal hysteresis, or the biomechanical response of the cornea to a brief air impulse, may be another promising phenotype. Corneal hysteresis is, in fact, a risk factor for visual field damage [6], [9] and the presence of glaucoma [10]. The pulsatility of choroidal blood flow, i.e. how choroidal blood flow changes with the pulse rate, is also a potential endophenotype for glaucoma given its relationship with glaucoma [11], [12]. Only phenotypes that are heritable are useful endophenotypes in the search for genes contributing to a complex disease.

Genetic research of glaucoma-related traits has mainly focused on intraocular pressure and central corneal thickness, which have been found to be heritable in multiple studies [13]–[21]. No prior studies have assessed the heritability of the pulsatility of choroidal blood flow. One twin study has examined corneal hysteresis and found it to be heritable [22]. Twin studies are very valuable study designs but replication of heritability estimates using other study designs is warranted. Indeed, heritability estimation from twin studies involves assumptions that are difficult to verify and may not be representative of the general population [23]. Hence, additional research on the heritability of glaucoma-related phenotypes is needed. Therefore, our goal was to assess the heritability of several glaucoma-related traits including the pulsatility of choroidal blood flow for the first time in order to propel the search for genes likely to contribute to the susceptibility of the optic nerve to glaucoma.

## Materials and Methods

### Study Population

Participants were recruited from the ophthalmology clinics of Maisonneuve-Rosemont Hospital in Montreal, Quebec. A sample of 47 Caucasian people without glaucoma was recruited between July 2008 and November 2009 from a community-based screening program or from advertising flyers displayed at community events. Each person in the sample recruited at least one first-degree relative without glaucoma to also participate in the study giving 96 people overall (47 families including 29 parent-offspring pairs and 21 sibling pairs). The absence of glaucoma was determined by normal optic nerve appearance, normal optic nerve head morphology using Heidelberg Retina Tomography (HRT-2) imaging, normal automated visual field using frequency doubling technology (FDT 24-2), normal intraocular pressure (<21mmHg using Goldman tonometry), and normal gonioscopy. People who had undergone prior ocular surgery were excluded. Other exclusion criteria included the presence of ocular disease, cloudy ocular media, or inability to cooperate for the exams. Written informed consent was obtained from all participants. The research conformed to the tenets of the Declaration of Helsinki. The study was approved by the Ethics Committee at Maisonneuve-Rosemont Hospital.

### Data Collection

Participants underwent an hour long examination by a single observer (DD) to collect the data. In order to minimize the influence of diurnal variation, almost all participants were examined between 9am and noon. The Pascal Dynamic Contour Tonometer (DCT) was used to give a measure of intraocular pressure (IOP) that is independent of corneal thickness (SMT Swiss Microtechnology AG, Port, Switzerland). This device is used at the slit lamp but contains a pressure transducer that is placed into contact with the cornea and a wireless digital recording device. The Pascal DCT gave a measure of IOP (IOPp) measured in mmHg. The Ocular Response Analyzer (ORA) (Reichert Ophthalmic Instruments, Depew, NY), a device used to direct a burst of air at the cornea, was used to derive two applanation pressure measurements, one during the depression of the cornea and another during the recovery [24]. The average of these two measures gives the Goldmann-correlated IOP (IOPg) while the difference between these two measures gives corneal hysteresis. The corneal hysteresis measure allows the calculation of a corneal compensated IOP (IOPcc), which is less affected by properties of the cornea than other measures of tonometry. The pulsatility of choroidal blood flow was measured in the sub-foveolar choroid using single point laser Doppler flowmetry (Oculix) [25]. Central corneal thickness was measured with an ultrasound pachymeter (Reichert Ophthalmic Instruments, Depew, NY) taking the mean of 50 consecutive measurements made during a single contact. Visual and ophthalmic exams were performed to confirm the absence of glaucoma or other ocular diseases and medical histories were reviewed for evidence of previous ocular surgery.

### Statistical Analysis

Data were used from one eye from each patient. The eye with the better visual acuity was used. If both eyes had the same visual acuity, the right eye was used. We performed heritability analyses using maximum likelihood variance components methods as implemented in the program SOLAR 26]. This approach can estimate heritability from families of arbitrary size and takes into account all relationships simultaneously by modeling the covariance among family members in terms of genetic proximity or kinship. Specifically, for a particular phenotype *y*, the value of *y* for individual *i* is modeled as 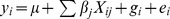, where *μ* is the mean of *y*, X*_ij_* is the *j*-th covariate with associated regression coefficient *β_j_*, g*_i_* is an additive genetic effect normally distributed with mean 0 and variance 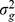, and e*_i_* is a random residual effect normally distributed with mean 0 and variance 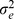. Any non-additive genetic (such as dominance) and unmeasured non-genetic effects (as well as random errors) are incorporated into e*_i_*. The narrow-sense heritability of the phenotype is estimated by the ratio of the variance attributable to additive genetic effects, 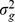, to the total phenotypic variance. We used likelihood-ratio tests to assess the significance of a parameter of interest by comparing the log-likelihood of the model in which the parameter is estimated to that of the model in which the parameter is fixed at zero 27]. We adjusted heritability estimates for age and sex. We also calculated heritability estimates from twice the regression coefficient of the offspring’s trait value on the parent’s trait value using the ROMPrev program 28] and from twice the sibling correlation coefficient 23]. Before performing the quantitative genetic analyses described above, we assessed the distributions of all traits and transformed them to approximate univariate normality when necessary. Only the pulsatility of choroidal blood flow was log-transformed. We assessed the impact of outliers on the estimates of heritability by examining the change in the estimates when extreme values were excluded. A p-value <0.05 was considered statistically significant. All analyses (except where noted above) were conducted using version 9.2 of the Statistical Analysis System programming language (SAS Institute, Cary, North Carolina, USA).

## Results

Out of the 47 families, there were 29 parent-offspring pairs and 21 sibling pairs. Forty-five families included only one pair (parent-offspring or sibling), one family included one parent and 2 children, and one family included a parent-offspring pair and the sibling of the parent. The mean age of the sample was 53 years old (SD = 15) and the sample was 60% female. Among parent-offspring pairs, the mean ages of the parents and offspring were 65 years old (SD = 11) and 39 years old (SD = 9), respectively. Parents were 76% female while offspring were 45% female. Among sibling pairs, the mean ages of the oldest and youngest sibling in the pair were 55 years old (SD = 13) and 51 years old (SD = 12), respectively. Older siblings were 67% female while younger siblings were 52% female. The mean values for the 7 glaucoma-related endophenotypes can be found in Table 1.

**Table 1:** Description of 7 glaucoma-related traits.

Device	Glaucoma-Related Traits	Mean (SD)
Ultrasound Pachymeter	Central Corneal Thickness (μM)	546.9 ± 34.9
Reichart Ocular Response Analyzer	IOPcc (mmHg)	16.6 ± 2.7
	IOPg (mmHg)	16.1 ± 3.5
	Corneal Hysteresis (mmHg)	10.4 ± 1.5
Pascal DCT	IOPp (mmHg)	16.8 ± 2.7
Oculix	Pulsatility of Blood Velocity (kH2)	0.35 ± 0.09
	Pulsatility of Blood Flow (H2U)	0.34 ± 0.10

IOPcc = corneal compensated intraocular pressure, IOPg = Goldmann-correlated intraocular pressure, IOPp = Pascal intraocular pressure.

In variance components models, we found high heritability estimates for central corneal thickness, corneal hysteresis, and the 3 measures of intraocular pressure (Table 2). The heritability for central corneal thickness was 0.68 (SE = 0.26). For corneal hysteresis, the likelihood was maximized at the upper boundary constraint of 1.0 for the heritability estimate, indicating high heritability but not allowing precise estimation for this trait. Heritability estimates derived separately from parent-offspring regression and sibling correlations also reached the upper boundary of 1.0. Heritability estimates for the three measures of intraocular pressure were 0.94 (SE = 0.22) for IOPg, 0.79 (SE = 0.24) for IOPcc, and 0.53 (SE = 0.26) for IOPp.

**Table 2:** Heritability of 7 glaucoma-related traits.

Device	Glaucoma-Related Traits	Unadjusted h^2^ (SE)	P-value	Adjusted* h^2^ (SE)	P-value
Ultrasound Pachymeter	Central Corneal Thickness	0.65 (0.26)	0.0097	0.68 (0.26)	0.0078
ORA	IOPcc	0.79 (0.24)	0.0021	0.79 (0.24)	0.0023
	IOPg	0.95 (0.22)	0.0002	0.94 (0.22)	0.0003
	Corneal Hysteresis	NA†		NA†	
Pascal	IOPp	0.55 (0.26)	0.0187	0.53 (0.26)	0.0239
Oculix	Pulsatility of Blood Velocity	0.02 (0.29)	0.4666	0.10 (0.32)	0.3813
	Pulsatility of Blood Flow‡	0.20 (0.28)	0.2409	0.16 (0.29)	0.2922

* adjusted for age and sex.† Estimate reached the upper boundary constraint of 1.‡ Data were log-transformed to achieve normality.ORA = Ocular Response Analyzer, IOPcc = corneal compensated intraocular pressure, IOPg = Goldmann-correlated intraocular pressure, IOPp = Pascal intraocular pressure.

We did not find that the pulsatility of choroidal blood flow measures were heritable as the velocity and flow heritability estimates were not significant and were less than 0.20 (Table 2).

Age and sex adjustment only slightly altered the heritability estimates (Table 2). In fact, age and sex did not explain a significant proportion of variance in the endophenotypes, except that corneal hysteresis was negatively associated with age (P = 0.001).

## Discussion

To our knowledge, no prior studies have examined the heritability of the pulsatility of choroidal blood flow. We did not find that the pulsatility of choroidal blood flow was heritable in our sample. Perhaps it is primarily determined by environmental factors. We do not think that poor reproducibility is the reason for the lack of heritability in our sample because we found in prior research of 35 people that the pulsatility of choroidal blood velocity and flow were reproducible (intraclass correlation coefficient = 0.79 and 0.80, respectively) (Unpublished data).

Our results are consistent with previous studies indicating that several other glaucoma-related traits are heritable. Out of the 3 IOP measures, we found that IOPg had the highest heritability (h^2^ = 0.95, SE = 0.22) compared to IOPcc (h^2^ = 0.79, SE = 0.24) or IOPp (h^2^ = 0.55, SE = 0.26). However, all three measures were strongly heritable. IOPg is affected by central corneal thickness, which itself is known to be heritable [6], [16], [18], [20], while IOPcc and IOPp are independent of central corneal thickness. Furthermore, IOPg and IOPcc were measured using the ORA, which is a non-contact tonometer, while IOPp was measured using the DCT, which is a contact tonometer. Perhaps IOP measurements from a contact tonometer are influenced by other corneal factors that are not heritable thereby resulting in a lower heritability than the non-contact tonometers.

Our data support a high heritability for corneal hysteresis as shown by the estimate reaching the boundary of 1.0. An estimate of heritability at the boundary is not precise as no standard error can be obtained. However, although we are not able to precisely determine the estimate of heritability for corneal hysteresis, we can conclude that the heritability is high, which agrees with the findings of Carbonaro *et al* in their study of 264 twin pairs (h^2^ = 0.77, 95% CI 0.70, 0.82) [22]. Further studies are needed to more precisely estimate the heritability of this trait in non-twin populations.

Our other results are consistent with prior literature in this research area. A study by Carbonaro *et al* also found that IOPg had the highest heritability compared to IOPp, IOPcc, and Goldmann applanation tonometry [13]. Prior studies of central corneal thickness also found it to be highly heritable [16], [18], [20]. For example, the Toh *et al* study, which examined 256 twin pairs, found a heritability of 0.95 [18]. Consistent with these heritability results, some genetic factors have been recently identified for central corneal thickness [29], [30] and IOP [31].

Our study uses parent-offspring and sibling pairs to confirm the results of prior twin studies, which is important given the limitations of twin studies, including generalizability. It should be noted, however, that heritability estimates are population-specific due to possible differences in total, environmental, or genetic variance components of the trait. Hence, comparison between populations should be made carefully. Our study is also novel in its examination of the heritability of some new potential glaucoma endophenotypes. Indeed, no other researchers have presented the heritability of the pulsatility of choroidal blood flow or velocity and only one other study has examined corneal hysteresis [22].

A limitation of our study is that we used a convenience sample of people without glaucoma from the community. Therefore, our sample is not fully representative of the population. We also restricted our sample to Caucasian individuals since genetic factors and heritability may differ according to ethnicity and we did not have enough other ethnicities to study them separately. In addition, we excluded individuals with any previous ocular surgery, which may have also affected the representativeness of our sample but was necessary since ocular surgery may affect our outcomes. Also, our study had limited precision with standard errors of 0.2 to 0.3. Although we had adequate power (80%) to detect heritability estimates greater or equal to 0.60 as significantly different than zero, we were underpowered to detect a heritability of 0.20 as we saw for the pulsatility of choroidal blood flow. However, low heritability estimates indicate that the trait may not be a good endophenotype for genetic studies. Given the large environmental component of the trait variance, it would be difficult to find genes explaining variation in this trait. More studies of the pulsatility of choroidal blood flow and velocity are needed to determine their utility as glaucoma endophenotypes.

Our study provides further support that corneal hysteresis, intraocular pressure, and central corneal thickness are heritable and may be suitable endophenotypes in the search for genes for open-angle glaucoma. Conversely, the pulsatility of choroidal blood velocity and flow were not heritable in this sample and may therefore be more influenced by environmental factors.
